# Supplementary material for: Effects of an aquatic protocol on electromyography activation and strength of lower limb muscles in blind women: A randomized controlled trial
Source: PLoS One. 2025 May 27;20(5):e0322395. doi: 10.1371/journal.pone.0322395 (PMC12111718; doi:10.1371/journal.pone.0322395)
Supplement: S2 File — (DOCX) [file pone.0322395.s002.docx]

**Title:** The Effects of Aquatic Exercise on Electromyographic Activation and Strength of Lower Limb Muscles in Blind Women: A Randomized Controlled Trial

**Brief Title:** Aquatic Exercise & Muscle Strength in Blind Women

**Study Overview**

**Brief Summary**

Visual impairment affects millions globally, significantly impacting daily functioning, especially in low-income countries. It often leads to social isolation, decreased mobility, and reduced physical activity, contributing to diminished physical fitness [1-3]. Individuals with visual impairments also experience deficits in postural control, muscle weakness, and impaired balance, increasing their risk of falls [1]. Muscle activation and strength are crucial for maintaining stability, and research indicates that lower limb muscle weakness correlates with greater center of pressure sways, particularly affecting the anterior and posterior regions of the lower leg and thigh [4].

Interventions aimed at enhancing postural control have shown positive outcomes for individuals with visual impairments [1, 5]. Among these, aquatic exercises are particularly promising due to their resistance against muscle movements. In water, the buoyancy and hydrostatic pressure provide support and stability while promoting strength, flexibility, and balance [6, 7]. Although the detrimental effects of visual impairment on postural control are recognized, the effectiveness of interventions like aquatic exercises on improving neuromuscular function is less understood. Research indicates that the absence of vision compromises postural control, elevating the risk of falls, as balance relies on visual, vestibular, and somatosensory systems.

Numerous studies have examined the impact of exercise programs on balance in individuals with visual impairments [1, 8]. For instance, a study found no impairments in static balance after whole-body vibration in individuals with congenital blindness [9]. The buoyant environment of water reduces the fear of falling and allows for greater movement freedom, making aquatic exercises effective for balance training [10]. Regular aquatic exercise programs have been shown to improve postural stability in visually impaired women by enhancing proprioceptive feedback and neuromuscular coordination.

**Aim**

This study aims to explore the effects of an aquatic exercise protocol on electromyography (EMG) activation and strength of lower limb muscles in women with visual impairments. We hypothesize that this protocol will increase EMG activation and muscle strength, thereby reducing fall risks and improving the quality of life. By targeting sensorimotor deficits, we anticipate that aquatic exercises will lead to significant improvements in functional abilities for individuals with visual impairments, facilitating the development of targeted rehabilitation strategies.

**Method and Materials**

Thirty blinded women were divided into two groups: Experimental (EX) and Control (CO). They underwent an eight-week aquatic protocol. EMG analysis was conducted in two directions (anterior-posterior and posterior-anterior) on a treadmill. The variables analyzed included MVIC% and onset time from four muscles: tibialis anterior, gastrocnemius medialis, rectus femoris, and biceps femoris. Additionally, data on muscle strength from ankle dorsiflexors, plantarflexors, knee flexors, and extensors were collected.

**Study design**

Single-blinded randomized controlled trial.

**Study setting**

Faculty of Sports Sciences, Shahid Bahonar University of Kerman, Iran.

**Participants**

The initial sample of participants was recruited from the Association of the Visually Impaired in Kerman City through collaboration with the organization's staff, from February 13, 2023, to April 10, 2023. Subsequently, 54 participants were enrolled for the initial assessment. Participants were randomly divided into two groups using the simple randomization method at a 1:1 ratio through the Random.org website: the experimental group (EX; n = 15) and the control group (CO; n = 15). After explaining the experimental protocols, each participant provided informed consent. The consent process included offering participants a written document in Braille or an audio recording of the consent form to ensure that they fully understood the study before consenting, in accordance with the Declaration of Helsinki and the ethical approval obtained from the ethical committee of Shahid Bahonar University of Kerman, Iran (ethical approval number: IR.KMU.REC.1395.598). The interventional program was conducted from May 4, 2023, to June 29, 2023.


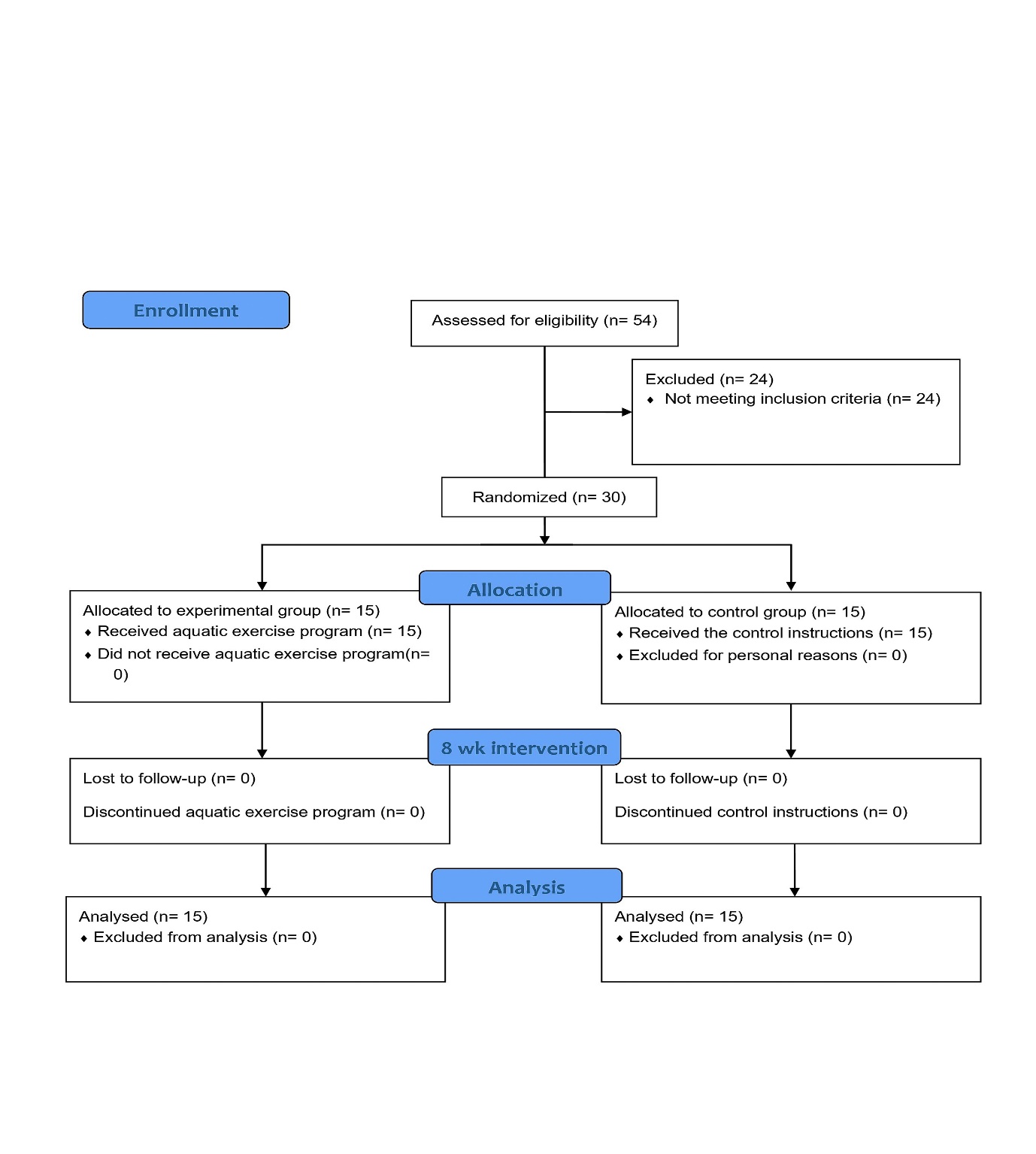


The exclusion criteria were:

1. Neurological disorders.
2. Auditory impairments.
3. History of vestibular system impairment.
4. Pathological symptoms.
5. Previous fractures or joint-related surgeries.
6. Lower limb diseases.

The inclusion criteria for this study were:

1. Female participants aged 18 to 40 years.
2. Normal body mass index (BMI) (between 18–24 kg/m², not overweight).
3. Blind individuals with:
4. Limited visual acuity of 20/600.
5. Restricted field of vision of 20°.
6. General health assessment was conducted using the General Health Questionnaire before inclusion. This approach allowed us to focus on overall health and suitability for the intervention rather than rigidly excluding based on GHQ scores alone.

**Testing Procedures**

Before conducting the main assessments, all participants underwent a familiarization session to ensure they understood the study's objectives and testing methods. Researchers provided verbal descriptions and tactile demonstrations to accommodate visually impaired participants. Pre-test assessments were conducted at the Faculty of Sports Sciences laboratory at Shahid Bahonar University in Kerman. Demographic data, including age, educational background, employment status, and marital status, were recorded. Participants were then introduced to the testing equipment and procedures, with verbal and tactile instructions to ensure comprehension. To maintain consistency, all tests were conducted under standardized conditions, including controlled lighting, temperature, and noise levels.

Electromyography (EMG) testing involved the use of surface bipolar electrodes (OT Bioelettronica, Italy) placed on the tibialis anterior (TA), gastrocnemius medialis (GM), rectus femoris (RF), and biceps femoris (BF), following the Surface Electromyography for the Non-Invasive Assessment of Muscles (SENIAM) guidelines [11]. Muscle activity was recorded using a 16-channel EMG amplifier (OT-Bioelettronica, Torino, Italy) with a sampling frequency of 2048 Hz. To normalize EMG signals, participants performed Maximum Voluntary Isometric Contraction (MVIC) tests. Additionally, a treadmill perturbation test was performed at a speed of 1.1 m/s, determined through pilot testing. Participants stood barefoot with arms crossed in a hip-width stance while forward and backward perturbations were induced. A safety harness was used to prevent falls during the test.

Muscle strength was assessed using a Lafayette Handheld Dynamometer (HHD) model-47904. The testing positions were standardized: ankle dorsiflexors and plantar flexors were assessed in a sitting position with a 90-degree hip angle, while knee extensors and flexors were evaluated with both hip and knee at 90 degrees [12]. A stabilization strap was used to secure the dynamometer and minimize measurement errors. Following the 8-week aquatic intervention, post-test assessments were conducted under identical conditions to ensure consistency and accuracy in data collection.

**Intervention**

The intervention consisted of an 8-week aquatic exercise program designed to improve neuromuscular function and lower limb strength in visually impaired women. Participants in the experimental group attended three supervised sessions per week, each lasting 60 minutes. An experienced instructor, trained in working with visually impaired individuals, led the sessions using verbal cues and tactile demonstrations to ensure proper execution of exercises. The intervention was structured into three phases: warm-up (5 minutes), main exercises (50 minutes), and cool-down (5 minutes).

The warm-up phase included walking at increasing speeds and stretching exercises targeting major muscle groups to prepare participants for the session. The main exercise phase incorporated various balance and strengthening exercises performed in water, including walking forward and backward with long strides, lateral walking, walking on tiptoes, and performing controlled trunk rotations. Participants also engaged in single-leg stance exercises, hip flexion and extension movements, and upper body resistance exercises in a half-squat position. Additionally, proprioceptive challenges such as standing on balance boards and walking on patterned surfaces were introduced. The difficulty of the exercises progressively increased throughout the intervention by modifying hand positions, increasing stride length, and incorporating more dynamic movements. The cool-down phase focused on gentle walking and stretching exercises to promote recovery and relaxation.

The control group did not participate in structured physical activity and continued their usual daily routines. However, to minimize bias, researchers conducted random weekly check-ins to monitor their activity levels and overall well-being. At the conclusion of the 8-week intervention, all participants underwent post-test assessments to evaluate changes in electromyography (EMG) activation, muscle onset time, and strength measurements, ensuring that the impact of the aquatic program was thoroughly analyzed.

**Statistical Analyses**

The sample size was determined based on previous similar studies and statistical formulas[13, 14]. using a confidence level of 95% and a power of 80% and specified effect size (0.46). The sample size calculation is expressed in Equation 1:

N = (Z1ـ α /2 + Z1ـ β)^2^ (S1^2^+ S2^2^) / (M1 – M2)^2^

The data collected for the research variables underwent statistical analysis using SPSS 26.0 for Windows (SPSS Inc., Chicago, IL, USA). The Shapiro-Wilk test was employed to confirm the normality of the distribution. A mixed-design analysis of variance (ANOVA) was conducted on all variables, with the time factor (pre-test and post-test) considered within subjects, and the group factor (EX and Co) treated as a between-subjects factor. If a significant interaction was found between factors, post hoc paired t-tests with Bonferroni correction for multiple comparisons were applied. Effect sizes (ES) for all parameters were calculated using partial η². In this context, a partial η² value of 0.02, 0.13, and 0.26 represented small, medium, and large effect sizes, respectively [15].

Additionally, to determine the statistical significance of differences between pre-test and post-test measurements, Cohen's d was calculated to assess the practical significance of these changes. For interpretation, a Cohen's d of 0.2, 0.5, and 0.8 or above represents small, medium, and large effect sizes, respectively [15]. The significance level was set at p < 0.05.

**References**

1. Salari, A., et al., *Effects of 8 weeks aquatic exercises on balance recovery strategies and center of pressure sways in blind women: A randomized controlled trial.* British Journal of Visual Impairment, 2024. **0**(0): p. 02646196241281254.

2. Schmid, M., et al., *Equilibrium during static and dynamic tasks in blind subjects: no evidence of cross-modal plasticity.* Brain, 2007. **130**(8): p. 2097-2107.

3. Augestad, L.B. and L. Jiang, *Physical activity, physical fitness, and body composition among children and young adults with visual impairments: A systematic review.* British Journal of Visual Impairment, 2015. **33**(3): p. 167-182.

4. Pizzigalli, L., et al., *Prevention of falling risk in elderly people: the relevance of muscular strength and symmetry of lower limbs in postural stability.* The Journal of Strength & Conditioning Research, 2011. **25**(2): p. 567-574.

5. Zhikai, Q., G. Zizhao, and W. Junsheng, *Effects of aerobic exercise on balance and mobility in individuals with visual impairment: a systematic review.* Disability and Rehabilitation, 2024. **46**(22): p. 5134-5143.

6. Torres-Ronda, L. and X.S. i del Alcázar, *The properties of water and their applications for training.* Journal of human kinetics, 2014. **44**: p. 237.

7. Abadi, F.H., et al., *A perspective on water properties and aquatic exercise for older adults.* International Journal of Aging Health and Movement, 2020. **2**(2): p. 1-10.

8. Rinehimer, M., et al., *Clinical Effectiveness of an Aquatic Exercise Program on Those With Visual Impairment: A Pilot Study.* The Journal of Aquatic Physical Therapy, 2024. **32**(1): p. 3-8.

9. Di Cagno, A., et al., *Acute effect of whole body vibration on postural control in congenitally blind subjects: a preliminary evidence.* Disability and Rehabilitation, 2018. **40**(22): p. 2632-2636.

10. Norouzi, Z., et al., *Comparing the Effects of Resistance and Hydrotherapic Exercises on Reaction Time and Balance in the Elderly suffering from Mild Cognitive Impairment.* Community Health Journal, 2021. **15**(1): p. 50-60.

11. Sacco, I.C., et al., *A method for better positioning bipolar electrodes for lower limb EMG recordings during dynamic contractions.* Journal of neuroscience methods, 2009. **180**(1): p. 133-137.

12. Katoh, M. and H. Yamasaki, *Comparison of Reliability of Isometric Leg Muscle Strength Measurements Made Using a Hand-Held Dynamometer with and without a Restraining Belt.* Journal of Physical Therapy Science, 2009. **21**(1): p. 37-42.

13. Hagstrom, A.D., K.A. Shorter, and P. Marshall, *Changes in unilateral upper limb muscular strength and EMG activity following a 16 week strength training intervention survivors of breast cancer.* J Strength Cond Res, 2017.

14. Jafarnezhadgero, A., et al., *Effect of Rehabilitation with Medicine Ball on Muscle Activity in Low Back Pain Patients during Walking.* Anesthesiology and Pain, 2023. **14**(2): p. 107-115.

15. Pallant, J., *SPSS survival manual: A step by step guide to data analysis using IBM SPSS*. 2020: Routledge.


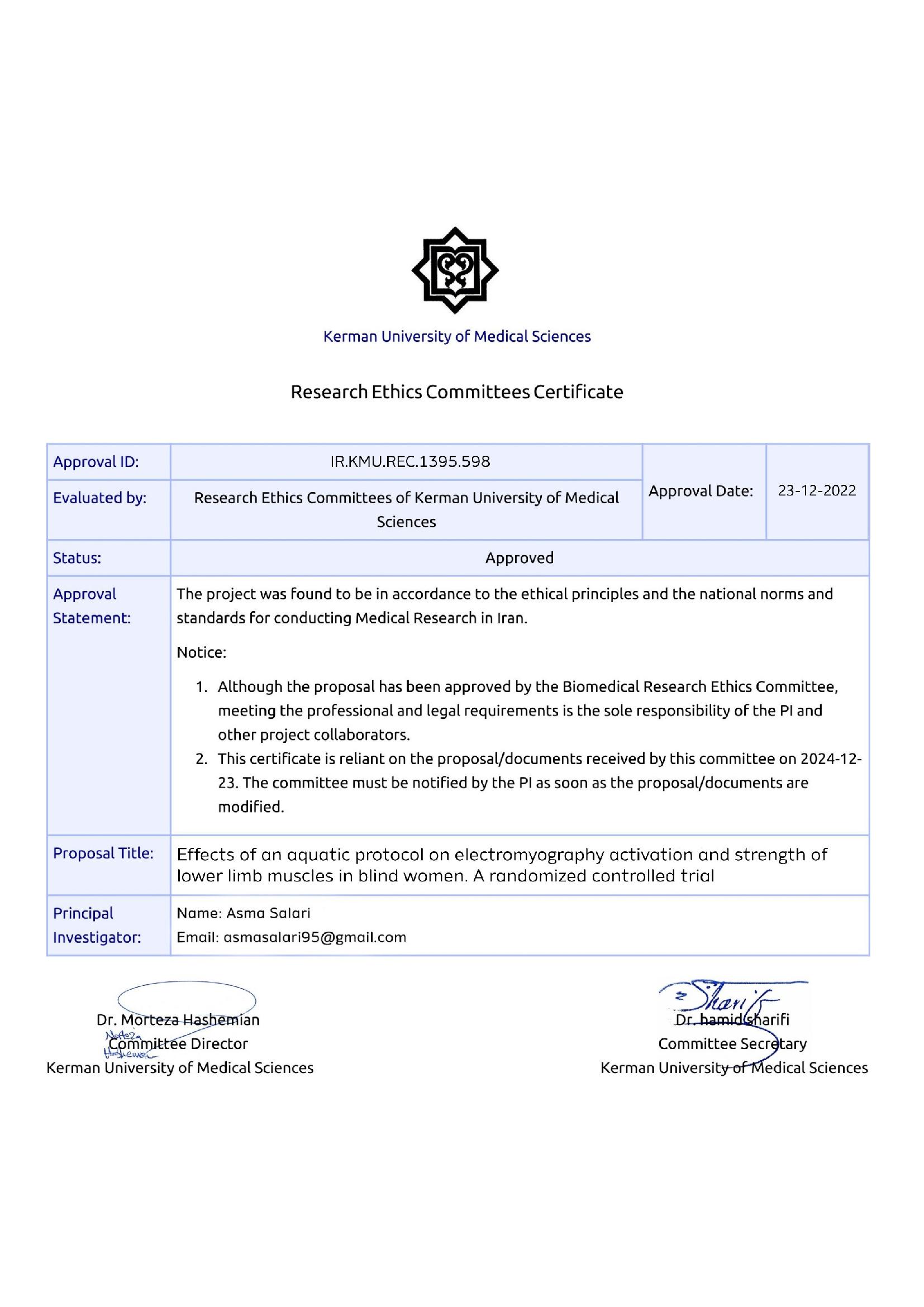


Dr Hamid Sharifi

Committee Secretary

Kerman University of Medical Sciences

Dr Morteza Hashemian

Committee Director

Kerman University of Medical Sciences

**Informed Consent Form**

**Title**

Effects of an aquatic protocol on electromyography activation and strength of lower limb muscles in blind women. A randomized controlled trial

*You are being invited to participate, as a participant, in a survey. Please read what follows carefully and ask me any questions you may have. After being clarified about the following information, if you agree to take part in this study, sign at the end of this document and initial the first page. The document will be signed in duplicate. One copy belongs to you and the other to the responsible researcher. In case of refusal, you will not suffer any penalty.*

This is research developed under the responsibility of the researcher Asma Salari, who is under the supervision/guidance of the main researcher Mansour Sahebozamani, from the University of Shahid Bahonar Kerman – Faculty of Sports Sciences – Department of Sports Injuries and Corrective Exercises.

- The purpose of this study is to evaluate the effects of a tailored aquatic exercise protocol on muscle activation and strength in women with visual impairments.
- Your participation will help in understanding how hydrotherapy-based exercises impact neuromuscular function and may contribute to improved rehabilitation strategies.
- If you participate, you will undergo physical tests before and after the study to measure muscle activation and strength. These tests include electromyography assessments and muscle strength measurements.
- The intervention consists of an 8-week aquatic exercise program, with three 60-minute sessions per week led by trained professionals.
- Participants will be randomly assigned to either the experimental group or the control group. The control group will continue with their normal activities without structured aquatic training.
- The procedures in this study follow international guidelines and have been designed to ensure participant safety.
- Possible minor discomfort or fatigue may occur during exercise sessions, but trained professionals will be available to assist you.
- If you feel any discomfort or wish to withdraw at any time, you have the right to discontinue participation without consequences.
- Your participation will contribute to scientific knowledge and rehabilitation strategies that may help improve muscle function and balance in visually impaired individuals.
- Your personal information and data collected during the study will remain confidential and used only for research purposes.
- You will not receive financial compensation for participation, nor will you incur any expenses related to the study. If any costs arise due to participation, you will be reimbursed.
- You have the right to withdraw from the study at any time without any penalty.
- At the end of the study, you may access your individual test results and choose to receive information on the overall research findings.
- Any questions or requests for clarifications can be directed to the supervisor responsible for the project: [Mansour Sahebozamani, Tel: (+98) 9133970521.
- For complaints and/or concerns regarding the ethical aspects of the research, contact the ethical committee of Shahid Bahonar University of Kerman, Tel: (+98) 034 33257447

“In view of the clarifications provided, I (participant's name)_______________________________, born on date ____**/**_**/**_____, I accept and agree to participate in the study ' The Effects of an Aquatic Protocol on Electromyography Activation and Strength of Lower Limb Muscles in Blind Women: A Randomized Controlled Trial', as a participant”.

| Volunteer's Full Name | Signature | Date |
| --- | --- | --- |
|  |  | / / |
| Responsible Researcher's full name | Signature | Date |
|  |  | / / |
