# Supplementary material for: Effects of an aquatic protocol on electromyography activation and strength of lower limb muscles in blind women: A randomized controlled trial
Source: PLoS One. 2025 May 27;20(5):e0322395. doi: 10.1371/journal.pone.0322395 (PMC12111718; doi:10.1371/journal.pone.0322395)
Supplement: S3 File — (DOCX) [file pone.0322395.s003.docx]

عنوان: تأثیر تمرینات ورزشی در آب بر فعال‌سازی الکترومایوگرافی و قدرت عضلات اندام تحتانی در زنان نابینا: یک کارآزمایی تصادفی کنترل‌شده

عنوان کوتاه: تمرینات آبی و قدرت عضلانی در زنان نابینا

مرور کلی مطالعه

خلاصه‌ای کوتاه

نقص بینایی میلیون‌ها نفر را در سراسر جهان تحت تأثیر قرار می‌دهد و تأثیر قابل‌توجهی بر عملکرد روزانه افراد، به‌ویژه در کشورهای کم‌درآمد، دارد. این مشکل می‌تواند منجر به انزوای اجتماعی، کاهش تحرک و کاهش فعالیت بدنی شود که در نهایت بر آمادگی جسمانی تأثیر منفی می‌گذارد [1-3]. افراد دارای نقص بینایی معمولاً در کنترل وضعیت بدن، قدرت عضلانی و تعادل دچار مشکل هستند که این امر خطر زمین خوردن آن‌ها را افزایش می‌دهد [1]. فعال‌سازی عضلات و قدرت آن‌ها نقش کلیدی در حفظ تعادل دارد و تحقیقات نشان می‌دهند ضعف عضلات اندام تحتانی با افزایش نوسانات مرکز فشار بدن مرتبط است که بیشتر نواحی جلویی و پشتی ساق و ران را تحت تأثیر قرار می‌دهد [4]. مطالعاتی که با هدف بهبود کنترل وضعیت بدن در افراد دارای نقص بینایی انجام شده‌اند، نتایج مثبتی را نشان داده‌اند [1, 5]. در میان این روش‌ها، تمرینات آبی به دلیل ایجاد مقاومت در برابر حرکات عضلانی، گزینه‌ای امیدوارکننده محسوب می‌شوند. در آب، نیروی شناوری و فشار هیدرواستاتیکی از بدن حمایت کرده و ضمن ایجاد ثبات، به بهبود قدرت، انعطاف‌پذیری و تعادل کمک می‌کنند [6, 7]. در حالی که تأثیر منفی نقص بینایی بر کنترل وضعیت بدن شناخته شده است، هنوز درک روشنی از تأثیر تمرینات آبی بر عملکرد عصبی-عضلانی وجود ندارد. مطالعات نشان داده‌اند که نبود بینایی باعث اختلال در کنترل وضعیت بدن شده و خطر سقوط را افزایش می‌دهد، زیرا تعادل بدن وابسته به سیستم‌های بینایی، وستیبولار و حسی-پیکری است. مطالعات متعددی تأثیر برنامه‌های ورزشی بر تعادل افراد دارای نقص بینایی را بررسی کرده‌اند [1, 8]. برای مثال، در یک مطالعه مشخص شد که پس از استفاده از ارتعاش کل بدن، تعادل ایستا در افراد نابینای مادرزادی دچار اختلال نمی‌شود [9]. محیط آبی به دلیل کاهش ترس از سقوط و امکان حرکت آزادانه‌تر، شرایط مناسبی برای تمرینات تعادلی فراهم می‌کند [10]. برنامه‌های منظم تمرینات آبی نشان داده‌اند که با تقویت بازخورد حس عمقی و هماهنگی عصبی-عضلانی، پایداری وضعیت بدن را در زنان نابینا بهبود می‌بخشند.

هدف

این مطالعه به بررسی تأثیر یک برنامه تمرینات آبی بر فعال‌سازی الکترومایوگرافی (EMG) و قدرت عضلات اندام تحتانی در زنان نابینا می‌پردازد. ما فرض می‌کنیم که این برنامه موجب افزایش فعال‌سازی الکترومایوگرافی و قدرت عضلانی شده و در نتیجه خطر سقوط را کاهش داده و کیفیت زندگی را بهبود می‌بخشد. با هدف قرار دادن نقص‌های حسی-حرکتی، انتظار می‌رود که تمرینات آبی منجر به بهبود قابل‌توجهی در توانایی‌های عملکردی افراد دارای نقص بینایی شود و زمینه‌ای برای توسعه استراتژی‌های توانبخشی هدفمند فراهم آورد.

مواد و روش‌ها

30 زن نابینا در دو گروه مداخله و کنترل تقسیم شدند و به مدت هشت هفته یک پروتکل تمرینی در آب را اجرا کردند. تحلیل الکترومایوگرافی (EMG) در دو جهت قدامی-خلفی و خلفی-قدامی بر روی تردمیل انجام شد. متغیرهای مورد بررسی شامل درصد حداکثر انقباض ارادی (MVIC%) و زمان شروع فعالیت عضلانی در چهار عضله تیبیالیس قدامی، گاستروکنمیوس داخلی، رکتوس فموریس و بایسپس فموریس بود. علاوه بر این، داده‌های مربوط به قدرت عضلانی از عضلات دورسی‌فلکسورها و پلانتارفلکسورهای مچ پا، فلکسورها و اکستنسورهای زانو جمع‌آوری شد.

طرح مطالعه

این پژوهش به‌صورت کارآزمایی تصادفی کنترل‌شده با طرح یک سویه‌کور انجام شد.

محل مطالعه

دانشکده علوم ورزشی، دانشگاه شهید باهنر کرمان، ایران

شرکت‌کنندگان

نمونه اولیه از اعضای انجمن نابینایان شهر کرمان با همکاری کارکنان این سازمان از 24/11/1401 تا 21/01/1402 جذب شد. در ابتدا، ۵۴ نفر برای ارزیابی اولیه ثبت‌نام کردند. سپس، شرکت‌کنندگان به‌صورت تصادفی ساده و با نسبت ۱:۱ از طریق وب‌سایت Random.org به دو گروه تقسیم شدند: گروه مداخله (15 نفر) و گروه کنترل (15 نفر). پس از توضیح کامل پروتکل‌های آزمایشی، رضایت‌نامه آگاهانه از تمامی شرکت‌کنندگان دریافت شد. این فرآیند شامل ارائه نسخه‌ای از فرم رضایت‌نامه به‌صورت بریل یا فایل صوتی بود تا اطمینان حاصل شود که شرکت‌کنندگان به‌طور کامل از جزئیات مطالعه آگاه هستند. این مطالعه مطابق با بیانیه هلسینکی و با تأییدیه کمیته اخلاق دانشگاه شهید باهنر کرمان (شماره تأییدیه اخلاق:
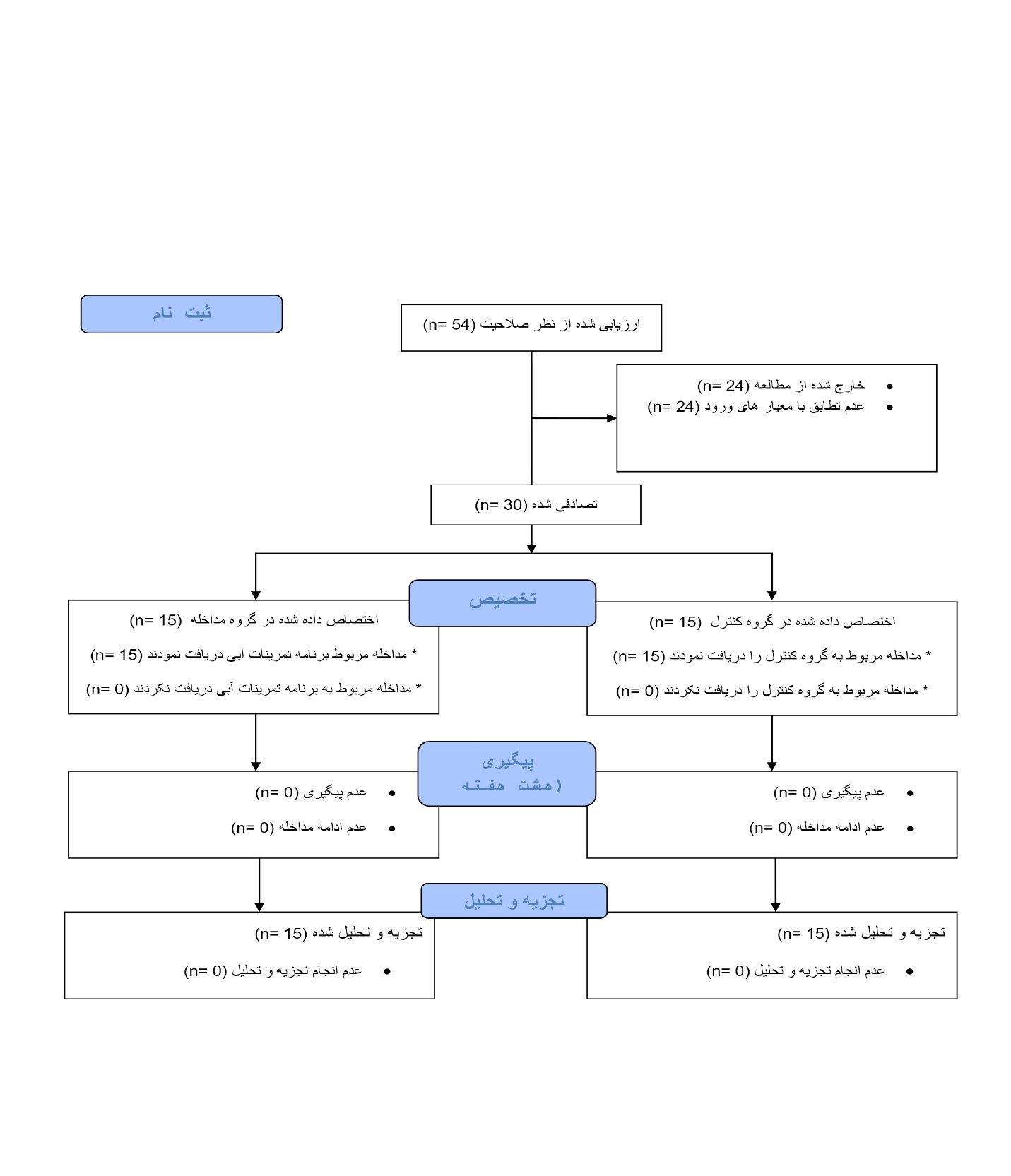
(IR.KMU.REC.1395.598) انجام شد. برنامه مداخله‌ای 14/02/1402 تا 08/04/1402 اجرا شد.

معیارهای خروج از مطالعه: شامل اختلالات عصبی، نقص شنوایی، سابقه مشکلات سیستم وستیبولار، علائم پاتولوژیک، سابقه شکستگی یا جراحی‌های مربوط به مفاصل، و بیماری‌های اندام تحتانی بود.

معیارهای ورود به مطالعه: شامل زنان در محدوده سنی ۱۸ تا ۴۰ سال، شاخص توده بدنی طبیعی (۱۸ تا ۲۴ کیلوگرم بر متر مربع) و نابینایان با تیزبینی محدود ۲۰/۶۰۰ و میدان دید ۲۰ درجه بود. همچنین، وضعیت سلامت عمومی شرکت‌کنندگان با استفاده از پرسش‌نامه سلامت عمومی ( (GHQ) ارزیابی شد تا اطمینان حاصل شود که آنها برای مداخله مناسب هستند .

روش اجرای پژوهش

قبل از انجام ارزیابی‌های اصلی، تمامی شرکت‌کنندگان در یک جلسه آشنایی شرکت کردند تا درک کاملی از اهداف و روش‌های مطالعه پیدا کنند. برای سازگاری با نیازهای افراد نابینا، پژوهشگران از توضیحات شفاهی و نمایش لمسی تجهیزات و روش‌های آزمون استفاده کردند. پیش‌آزمون‌ها در آزمایشگاه دانشکده علوم ورزشی دانشگاه شهید باهنر کرمان انجام شد. اطلاعات دموگرافیک شامل سن، سطح تحصیلات، وضعیت شغلی و تأهل ثبت شد. سپس شرکت‌کنندگان با تجهیزات و روش‌های آزمون آشنا شدند و دستورالعمل‌ها به‌صورت شفاهی و لمسی ارائه گردید. برای حفظ یکپارچگی داده‌ها، تمامی آزمون‌ها در شرایط استاندارد شامل نور، دما و سطح نویز کنترل‌شده انجام شد. برای ثبت فعالیت عضلانی، از الکترودهای دو قطبی سطحی (OT Bioelettronica، ایتالیا) استفاده شد که بر اساس دستورالعمل‌های SENIAM بر روی عضلات تیبیالیس قدامی، گاستروکنمیوس داخلی، رکتوس فموریس و بایسپس فموریس قرار گرفتند. داده‌های EMG با استفاده از تقویت‌کننده ۱۶ کاناله EMG OT-Bioelettronica)، تورین، ایتالیا) با فرکانس نمونه‌برداری ۲۰۴۸ هرتز ثبت شد [11]. برای نرمال‌سازی سیگنال‌های EMG، شرکت‌کنندگان آزمون حداکثر انقباض ایزومتریک ارادی را انجام دادند. همچنین، آزمون اغتشاش بر روی تردمیل با سرعت ۱.۱ متر بر ثانیه (تعیین‌شده بر اساس تست پایلوت) اجرا شد. شرکت‌کنندگان در حالت ایستاده بدون کفش، با دستان ضربدری روی سینه و پاها در عرض لگن قرار گرفتند و اغتشاشات رو به جلو و عقب ایجاد شد. برای جلوگیری از سقوط، از یک کمربند ایمنی که به سقف متصل بود، استفاده گردید [11]. قدرت عضلانی با استفاده از دینامومتر دستی (Lafayette HHD، مدل ۴۷۹۰۴) اندازه‌گیری شد. موقعیت‌های آزمون استانداردسازی شده بود: قدرت دورسی‌فلکسورها و پلانتارفلکسورهای مچ پا در حالت نشسته با زاویه ۹۰ درجه لگن ارزیابی شد. همچنین، قدرت فلکسورها و اکستنسورهای زانو در حالت نشسته با زاویه۹۰ درجه در لگن و زان اندازه‌گیری شد. برای کاهش خطاهای اندازه‌گیری، از بندهای تثبیت‌کننده برای نگه‌داشتن دینامومتر استفاده شد [12]. پس از هشت هفته مداخله در آب، پس‌آزمون‌ها تحت شرایط یکسان با پیش‌آزمون انجام شد تا دقت و یکپارچگی داده‌ها تضمین گردد.

مداخله

این مطالعه شامل یک برنامه تمرینات آبی ۸ هفته‌ای بود که با هدف بهبود عملکرد عصبی-عضلانی و افزایش قدرت اندام تحتانی در زنان دارای اختلال بینایی طراحی شد. گروه آزمایشی سه جلسه تحت نظارت در هفته، هر جلسه به مدت ۶۰ دقیقه، در این برنامه شرکت کردند. یک مربی باتجربه که آموزش‌های لازم برای کار با افراد دارای اختلال بینایی را دیده بود، جلسات را هدایت می‌کرد. برای اطمینان از اجرای صحیح تمرینات، از راهنمایی‌های کلامی و نمایش‌های لمسی استفاده شد.

برنامه تمرینی شامل سه مرحله بود: گرم‌کردن (۵ دقیقه)، تمرینات اصلی (۵۰ دقیقه) و سرد کردن (۵ دقیقه).

مرحله گرم‌کردن شامل راه رفتن با سرعت‌های متغیر و انجام حرکات کششی برای گروه‌های عضلانی اصلی بود تا بدن شرکت‌کنندگان برای تمرین آماده شود.

مرحله تمرینات اصلی شامل تمرینات مختلف تعادلی و قدرتی در آب بود، از جمله:

- **راه رفتن رو به جلو و عقب با گام‌های بلند**

- **راه رفتن به طرفین**

- **راه رفتن روی پنجه پا**

- **چرخش‌های کنترل‌شده‌ی تنه**

علاوه بر این، شرکت‌کنندگان تمریناتی مانند **ایستادن روی یک پا، حرکات خم و باز کردن مفصل ران، و تمرینات مقاومتی نیمه‌اسکوات برای بالاتنه** را انجام دادند. برای بهبود حس عمقی، چالش‌هایی مانند ایستادن روی تخته‌های تعادلی و راه رفتن روی سطوح الگو‌دار اضافه شد. شدت تمرینات به‌صورت تدریجی افزایش یافت، از جمله با تغییر موقعیت دست‌ها، افزایش طول گام‌ها و افزودن حرکات پویا.

مرحله سرد کردن: شامل راه رفتن آرام و حرکات کششی ملایم برای کمک به بازیابی و آرام‌سازی بدن بود.

گروه کنترل در هیچ فعالیت ورزشی ساختاریافته‌ای شرکت نکردند و به روال روزمره خود ادامه دادند. با این حال، برای کاهش سوگیری، پژوهشگران به‌صورت هفتگی وضعیت فعالیت و سلامت عمومی این شرکت‌کنندگان را بررسی کردند. پس از پایان دوره ۸ هفته‌ای، آزمون‌های پس‌آزمون برای تمامی شرکت‌کنندگان انجام شد تا تغییرات در فعال‌سازی الکترومایوگرافی (EMG) زمان شروع عضلانی و میزان قدرت بررسی شود و تأثیر برنامه ورزشی آبی به‌طور دقیق تحلیل گردد.

تحلیل‌های آماری

تعیین حجم نمونه بر اساس مطالعات پیشین و فرمول‌های آماری انجام شد [13, 14]. با در نظر گرفتن سطح اطمینان ۹۵٪، توان آماری ۸۰٪ و اندازه اثر ۰.۴۶، حجم نمونه محاسبه شد. فرمول محاسبه حجم نمونه به‌صورت زیر است:

N = (Z1ـ α /2 + Z1ـ β)^2^ (S1^2^+ S2^2^) / (M1 – M2)^2^

برای تحلیل داده‌های پژوهش از نرم‌افزار SPSS نسخه ۲۶.۰ (SPSS Inc., Chicago, IL, USA) استفاده شد. آزمون شاپیرو-ویلک جهت بررسی نرمال بودن توزیع داده‌ها به کار رفت. برای تحلیل متغیرها، از تحلیل واریانس با طرح ترکیبی (ANOVA) استفاده شد که در آن عامل زمان (پیش‌آزمون و پس‌آزمون) به‌عنوان یک متغیر درون‌گروهی و عامل گروه (مداخله و کنترل) به‌عنوان یک متغیر بین‌گروهی در نظر گرفته شد.

در صورت وجود تعامل معنادار بین متغیرها، آزمون t همبسته پس از مداخله با اصلاح بونفرونی برای مقایسه‌های چندگانه اجرا شد. همچنین، برای محاسبه اندازه اثر partial η² استفاده شد. در این تحلیل:

- مقدار ۰.۰۲ نشان‌دهنده اندازه اثر کوچک،

- مقدار ۰.۱۳ نشان‌دهنده اندازه اثر متوسط،

- و مقدار ۰.۲۶ نشان‌دهنده اندازه اثر بزرگ بود [15].

علاوه بر آن، برای تعیین معناداری آماری تفاوت بین پیش‌آزمون و پس‌آزمون، Cohen’s d محاسبه شد تا معناداری عملی این تغییرات مشخص شود. در این تحلیل:

- مقدار ۰.۲ نشان‌دهنده اندازه اثر کوچک،

- مقدار ۰.۵ اندازه اثر متوسط،

- و مقدار ۰.۸ یا بالاتر نشان‌دهنده اندازه اثر بزرگ در نظر گرفته شد [15]. سطح معناداری در تمامی تحلیل‌ها p < 0.05 تعیین شد.

منابع

1. Salari, A., et al., *Effects of 8 weeks aquatic exercises on balance recovery strategies and center of pressure sways in blind women: A randomized controlled trial.* British Journal of Visual Impairment, 2024. **0**(0): p. 02646196241281254.

2. Schmid, M., et al., *Equilibrium during static and dynamic tasks in blind subjects: no evidence of cross-modal plasticity.* Brain, 2007. **130**(8): p. 2097-2107.

3. Augestad, L.B. and L. Jiang, *Physical activity, physical fitness, and body composition among children and young adults with visual impairments: A systematic review.* British Journal of Visual Impairment, 2015. **33**(3): p. 167-182.

4. Pizzigalli, L., et al., *Prevention of falling risk in elderly people: the relevance of muscular strength and symmetry of lower limbs in postural stability.* The Journal of Strength & Conditioning Research, 2011. **25**(2): p. 567-574.

5. Zhikai, Q., G. Zizhao, and W. Junsheng, *Effects of aerobic exercise on balance and mobility in individuals with visual impairment: a systematic review.* Disability and Rehabilitation, 2024. **46**(22): p. 5134-5143.

6. Torres-Ronda, L. and X.S. i del Alcázar, *The properties of water and their applications for training.* Journal of human kinetics, 2014. **44**: p. 237.

7. Abadi, F.H., et al., *A perspective on water properties and aquatic exercise for older adults.* International Journal of Aging Health and Movement, 2020. **2**(2): p. 1-10.

8. Rinehimer, M., et al., *Clinical Effectiveness of an Aquatic Exercise Program on Those With Visual Impairment: A Pilot Study.* The Journal of Aquatic Physical Therapy, 2024. **32**(1): p. 3-8.

9. Di Cagno, A., et al., *Acute effect of whole body vibration on postural control in congenitally blind subjects: a preliminary evidence.* Disability and Rehabilitation, 2018. **40**(22): p. 2632-2636.

10. Norouzi, Z., et al., *Comparing the Effects of Resistance and Hydrotherapic Exercises on Reaction Time and Balance in the Elderly suffering from Mild Cognitive Impairment.* Community Health Journal, 2021. **15**(1): p. 50-60.

11. Sacco, I.C., et al., *A method for better positioning bipolar electrodes for lower limb EMG recordings during dynamic contractions.* Journal of neuroscience methods, 2009. **180**(1): p. 133-137.

12. Katoh, M. and H. Yamasaki, *Comparison of Reliability of Isometric Leg Muscle Strength Measurements Made Using a Hand-Held Dynamometer with and without a Restraining Belt.* Journal of Physical Therapy Science, 2009. **21**(1): p. 37-42.

13. Hagstrom, A.D., K.A. Shorter, and P. Marshall, *Changes in unilateral upper limb muscular strength and EMG activity following a 16 week strength training intervention survivors of breast cancer.* J Strength Cond Res, 2017.

14. Jafarnezhadgero, A., et al., *Effect of Rehabilitation with Medicine Ball on Muscle Activity in Low Back Pain Patients during Walking.* Anesthesiology and Pain, 2023. **14**(2): p. 107-115.

15. Pallant, J., *SPSS survival manual: A step by step guide to data analysis using IBM SPSS*. 2020: Routledge.


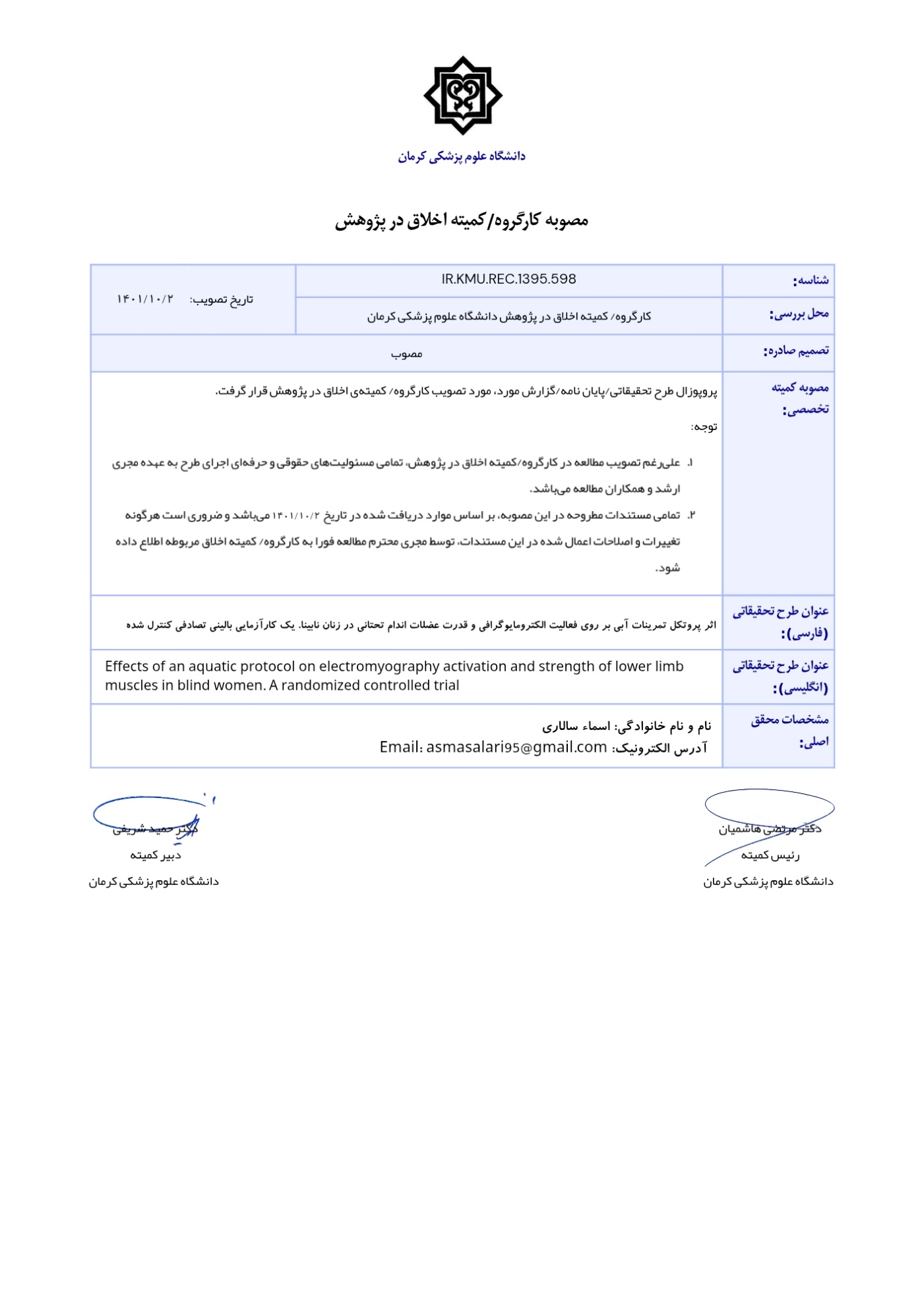


دکتر حمید شریفی

دبیر کمیته

دانشگاه علوم پزشکی کرمان

دکتر مرتضی هاشمیان

مدیر کمیته

دانشگاه علوم پزشکی کرمان

**فرم رضایت‌نامه آگاهانه**

عنوان پژوهش:

بررسی تأثیر یک پروتکل تمرینات آبی بر فعال‌سازی الکترومایوگرافی و قدرت عضلات اندام تحتانی در زنان نابینا: یک کارآزمایی تصادفی کنترل‌شده

شما به عنوان یکی از شرکت‌کنندگان، برای شرکت در این پژوهش دعوت شده‌اید. لطفاً متن زیر را با دقت مطالعه کنید و در صورت داشتن هرگونه سؤال، آن را مطرح نمایید. پس از دریافت توضیحات لازم، در صورتی که مایل به شرکت در این مطالعه هستید، لطفاً این فرم را امضا نمایید. این فرم در دو نسخه تنظیم می‌شود؛ یک نسخه نزد شما خواهد ماند و نسخه دیگر در اختیار پژوهشگر مسئول قرار خواهد گرفت. در صورت عدم موافقت با شرکت در پژوهش، هیچ‌گونه پیامد یا جریمه‌ای متوجه شما نخواهد شد.

این پژوهش تحت مسئولیت اسماء سالاری و با نظارت دکتر منصور صاحب‌الزمانی، استاد راهنما از دانشگاه شهید باهنر کرمان – دانشکده علوم ورزشی – گروه آسیب‌شناسی ورزشی و حرکات اصلاحی انجام می‌شود.

- هدف از این پژوهش بررسی تأثیر یک پروتکل تمرینات آبی بر فعال‌سازی عضلانی و قدرت عضلات در زنان دارای اختلال بینایی است.

- مشارکت شما به درک بهتر تأثیر تمرینات مبتنی بر آب بر عملکرد عصبی-عضلانی کمک می‌کند و می‌تواند به بهبود راهکارهای توان‌بخشی منجر شود.

- در صورت شرکت در مطالعه، شما پیش از شروع و پس از پایان دوره، تحت آزمون‌های فیزیکی قرار خواهید گرفت تا میزان فعالیت عضلانی و قدرت عضلات شما اندازه‌گیری شود. این آزمون‌ها شامل ارزیابی‌های الکترومایوگرافی (EMG) و اندازه‌گیری قدرت عضلانی هستند.

- برنامه مداخله‌ای شامل ۸ هفته تمرینات آبی است که هفته‌ای ۳ جلسه ۶۰ دقیقه‌ای و تحت نظارت متخصصان آموزش‌دیده برگزار خواهد شد.

- شرکت‌کنندگان به‌صورت تصادفی به دو گروه مداخله و کنترل تقسیم می‌شوند. گروه آزمایشی در برنامه تمرینات آبی شرکت می‌کند، در حالی که گروه کنترل بدون شرکت در تمرینات، به فعالیت‌های روزمره خود ادامه خواهد داد.

- تمامی مراحل پژوهش مطابق با استانداردهای بین‌المللی طراحی شده‌اند تا ایمنی و سلامت شرکت‌کنندگان تضمین شود.

- ممکن است در حین انجام تمرینات، احساس خستگی یا ناراحتی خفیف داشته باشید، اما متخصصان حاضر در جلسات، برای کمک و راهنمایی شما حضور خواهند داشت.

- در هر زمان که احساس ناراحتی کنید، می‌توانید از ادامه مطالعه انصراف دهید و این تصمیم هیچ‌گونه پیامد منفی برای شما نخواهد داشت.

- مشارکت شما در این پژوهش به توسعه دانش علمی و استراتژی‌های توان‌بخشی که ممکن است به بهبود عملکرد عضلانی و تعادل در افراد دارای اختلال بینایی کمک کند، یاری خواهد رساند.

- اطلاعات شخصی شما و داده‌های جمع‌آوری‌شده کاملاً محرمانه خواهند بود و فقط برای اهداف تحقیقاتی مورد استفاده قرار خواهند گرفت.

- شرکت در این پژوهش هیچ‌گونه هزینه‌ای برای شما نخواهد داشت و هیچ پرداخت مالی نیز به شما تعلق نخواهد گرفت. در صورتی که هرگونه هزینه‌ای به دلیل شرکت در این پژوهش متحمل شوید، این هزینه به شما بازپرداخت خواهد شد.

- شما در هر زمان، بدون هیچ‌گونه جریمه‌ای، حق انصراف از مطالعه را دارید.

- در پایان پژوهش، شما می‌توانید به نتایج فردی خود دسترسی داشته باشید و در صورت تمایل، اطلاعاتی درباره یافته‌های کلی تحقیق دریافت کنید.

- برای هرگونه سؤال یا درخواست توضیحات بیشتر، می‌توانید با استاد راهنمای پروژه تماس بگیرید:

دکتر منصور صاحب‌الزمانی، تلفن: 9133970521(+98)

- در صورت داشتن هرگونه شکایت یا نگرانی درباره جنبه‌های اخلاقی این پژوهش، می‌توانید با کمیته اخلاق دانشگاه شهید باهنر کرمان تماس بگیرید:

تلفن: 034 33257447(+98)

«با توجه به توضیحات ارائه‌شده، اینجانب (نام شرکت‌کننده)_______________________________، متولد ____/__/_____، با آگاهی و رضایت کامل، موافقت خود را برای شرکت در پژوهش "بررسی تأثیر یک پروتکل تمرینات آبی بر فعال‌سازی الکترومایوگرافی و قدرت عضلات اندام تحتانی در زنان نابینا: یک کارآزمایی تصادفی کنترل‌شده" اعلام می‌دارم.»

| تاریخ | امضا | نام و نام خانوادگی داوطلب |
| --- | --- | --- |
|  |  |  |
| تاریخ | امضا | نام و نام خانوادگی پژوهشگر مسئول |
|  |  |  |
